# Gene therapy for non-obstructive azoospermia: therapeutic avenues and translational pathway to clinical application

**DOI:** 10.3389/fgeed.2026.1921175

**Published:** 2026-09-30

**Authors:** Man-Xi Jiang, Tao Jing, Huai L. Feng, Yan Zhu

**Affiliations:** 1 Center for Reproductive Medicine, The Affiliated Guangdong Second Provincial General Hospital of Jinan University, Guangzhou, China; 2 New York Fertility Center, New York-Prebyterian Healthcare System Affiliate Weill Cornell Medical College, New York, NY, United States; 3 Department of Immunology, University of Pittsburgh, Pittsburgh, PA, United States

**Keywords:** CRISPR/Cas9, gene therapy, lipid nanoparticles, male infertility, mRNA delivery, non-obstructive azoospermia, spermatogenesis

## Abstract

Non-obstructive azoospermia (NOA) represents the most severe male infertility. Although microsurgical testicular sperm extraction combined with intracytoplasmic sperm injection offers opportunities, approximately 50% of NOA patients still fail to obtain sperm for clinical treatments. Fortunately, the emergence of precision medicine, particularly advances in gene therapy, has opened new therapeutic avenues for NOA in preclinical studies, demonstrating proof-of-concept in animal models. This review examines current progress in NOA gene therapy. Firstly, three principal therapeutic strategies: lipid nanoparticle-mediated mRNA delivery for transient protein replacement, CRISPR-based gene editing for permanent correction, and stem cell-based approaches combined with genetic modification are comparatively analyzed. Subsequently, particular emphasis is placed on recent breakthroughs in testicular mRNA delivery that have successfully restored spermatogenesis in NOA mouse models, resulting in viable offspring. Furthermore, the delivery technologies including adeno-associated viral vectors, and non-viral methods are critically evaluated to assess their respective advantages, limitations, and suitability for different testicular cell types. The review also addresses critical safety considerations, such as off-target effects, germline transmission risks, and epigenetic consequences. Finally, we discuss the translational roadmap toward clinical application, and how the obstacles including delivery optimization, long-term safety validation, and the regulatory pathways to affect the transition from preclinical proof-of-concept to first-in-human trials.

## Introduction

Infertility represents a major global health challenge, affecting approximately 17.5% of the reproductive-age population, equivalent to one in six individuals worldwide (Feng et al., 2025), while male factors contribute to nearly half of all infertility cases, underscoring the critical need for continued research in this area (Krausz, 2011; Tou et al., 2017). Among male infertility disorders, azoospermia is the most severe phenotype, affecting 10%–15% of infertile men (Piechk et al., 2024). For azoospermia patients, the psychological toll of this condition is profound, with affected individuals frequently report diminished quality of life and experience significant emotional distress, highlighting the urgent clinical and psychosocial imperatives for effective therapeutic interventions (Wu et al., 2023).

Azoospermia is broadly categorized into two primary forms: obstructive azoospermia (OA), which results from physical occlusion of the reproductive tract, and non-obstructive azoospermia (NOA), which arises from intrinsic impairment of spermatogenesis (Wosnitzer et al., 2014). NOA accounts for approximately 60% of all azoospermia cases and encompasses a heterogeneous spectrum of histopathological phenotypes, including Sertoli cell-only syndrome, germ cell maturation arrest, and hypospermatogenesis (Practice Committee of the American Society for Reproductive Medicine, 2018; Mostafa et al., 2025). Compared with OA, NOA poses substantially greater therapeutic challenges, as its etiology is rooted in multifaceted disturbances involving the testicular microenvironment, intrinsic germ cell defects, and the complex regulatory networks that orchestrate spermatogenesis (Wyrwoll et al., 2024).

For NOA patients who desire biological parenthood, microdissection testicular sperm extraction (micro-TESE) followed by intracytoplasmic sperm injection (ICSI) currently represents the standard treatment (Schlegel, 2009; Liu et al., 2024). At present, the overall sperm retrieval rates (SRRs) of micro-TESE range from 40% to 60%, varying by underlying etiology (Be et al., 2015; Corona et al., 2019; Caroppo et al., 2019). However, for patients with NOA caused by specific defects in meiosis-related genes that lead to complete maturation arrest, the probability of sperm retrieval via micro-TESE is extremely low (Weedin et al., 2011). These patients often face limited options, such as donor sperm or adoption, which present psychosocial and ethical challenges in certain cultural contexts, therefore these persistent gaps underscore the urgent need for novel therapeutic strategies (Sikiru et al., 2025).

The emergence of precision medicine has transformed therapeutic paradigms across numerous medical disciplines. For NOA, precision medicine holds particular promise for several reasons. First, gene sequencing technologies enable the identification of numerous genes essential for human spermatogenesis (Oud et al., 2019; Houston et al., 2021). Second, the genetic architecture of NOA is increasingly understood, and monogenic cases are increasingly being identified (Nagirnaja et al., 2022; Kasak and Laan, 2021). Third, based on genetic diagnoses, gene therapy capable of delivering corrective genetic material to specific testicular cells is being developed to offer potential for NOA treatment (Sikiru et al., 2025).

This review provides a comprehensive overview of the evolving landscape of gene therapy for NOA, focusing on three principal strategies: mRNA replacement via lipid nanoparticle (LNP) delivery, CRISPR-based genome editing for genetic correction, and stem cell-based approaches coupled with *ex vivo* genetic modification. Each modality is critically evaluated with respect to its scientific rationale, preclinical evidence, safety profile, and translational potential. Particular emphasis is placed on recent breakthroughs in the restoration of spermatogenesis in animal models, which represent a pivotal milestone on the path toward clinical application. In addition, this review addresses the unique anatomical, physiological, and immunological hurdles associated with targeted gene delivery to the testis, and culminates in a forward-looking roadmap for the clinical translation of gene therapy for NOA.

## Genetic basis of gene therapy for non-obstructive azoospermia

### Genomic landscape of NOA-associated genes

Recent advances in genomic technologies have substantially expanded our understanding of the genetic landscape underlying spermatogenesis (Tüttelmann et al., 2018). Early approaches, which relied primarily on candidate gene screening and familial linkage analyses, have largely been superseded by large-scale exome and genome sequencing in cohorts of men with idiopathic NOA (Lopes et al., 2013).

Currently, monogenic causes are estimated to account for 20%–40% of NOA cases, and this figure is anticipated to rise as additional causative genes are identified and functionally validated (Gershoni et al., 2017; Xavier et al., 2021). The Online Mendelian Inheritance in Man (OMIM) database currently catalogs over 100 genes associated with human male infertility (Hamosh et al., 2002), with numerous additional candidates characterized in animal models, particularly through forward genetic screens and targeted knockout studies in mice (Singh and Schimenti, 2025). Transcriptomic analyses of human spermatogenesis have revealed that these genes are involved in a remarkably wide array of biological processes, encompassing germ cell development, meiosis, chromatin remodeling, and hormonal signaling (Jan et al., 2017), highlighting the multifaceted nature of genetic regulation in male fertility (Figure 1).

**FIGURE 1:**
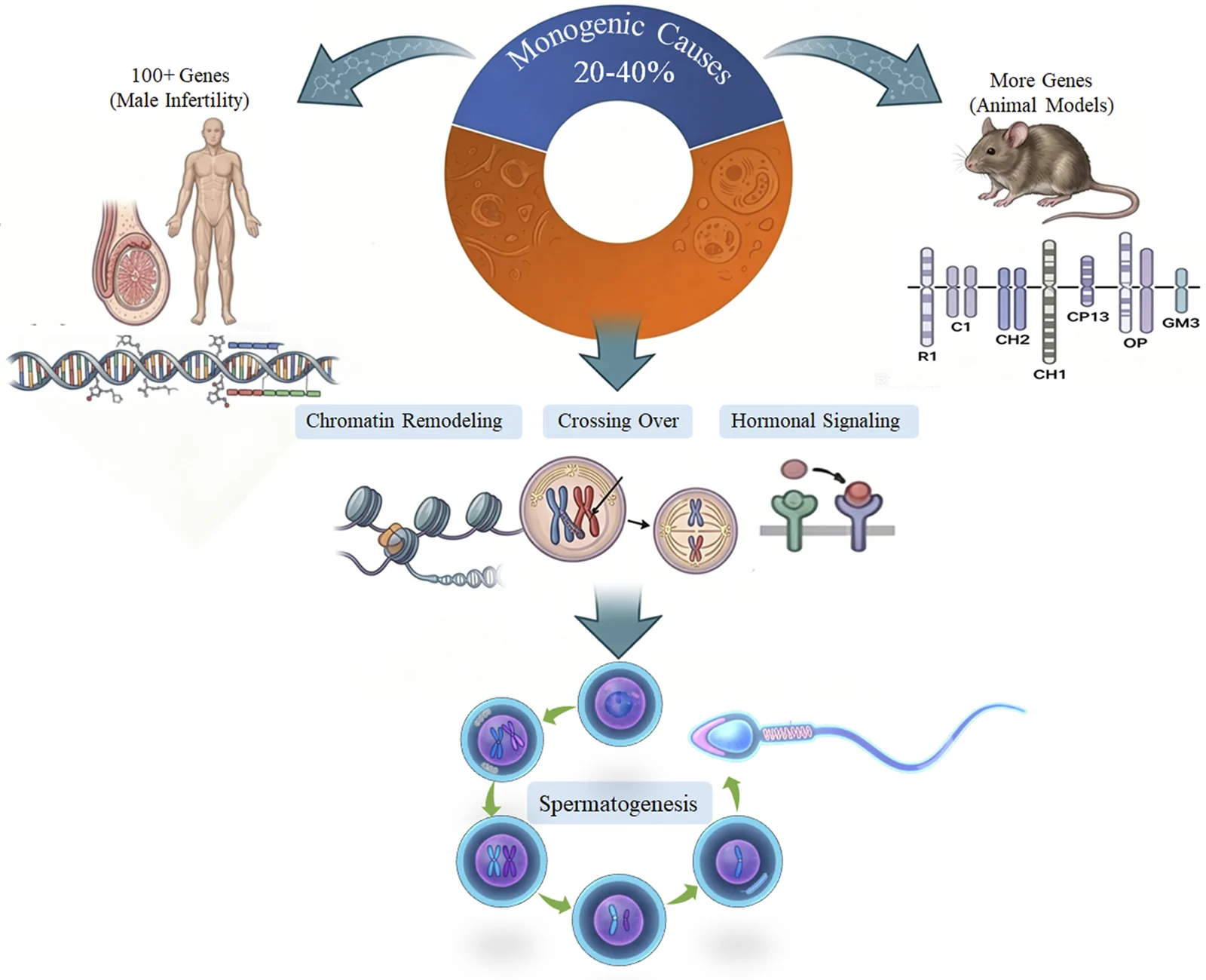
Overview of Genetic and Cellular Mechanisms Underlying Male Infertility. This figure summarizes key biological processes and genetic contributors associated with male infertility. Over 100 genes have been implicated, with monogenic causes accounting for an estimated 20%–40% of cases. Additional genes have been identified through animal models. The schematic highlights several critical cellular events, including chromatin remodeling, crossing over during meiosis, hormonal signaling, metaphase II arrest, and germ cell development. This integrative view emphasizes the multifactorial nature of male infertility, involving both hormonal and meiotic disruptions at the genetic and cellular levels.

## Meiotic genes and spermatogenic arrest

Meiosis-related genes currently represent the most promising targets for gene therapy in male infertility (Handel and Schimenti, 2010). Mutations affecting these genes typically result in early spermatogenic arrest at the pachytene stage, a critical phase during which homologous chromosomes synapse and undergo recombination (Ozturk, 2023) (Figure 2). In such cases, surgical sperm retrieval is universally ineffective, as haploid germ cells are entirely absent (Wyrwoll et al., 2020).

**FIGURE 2:**
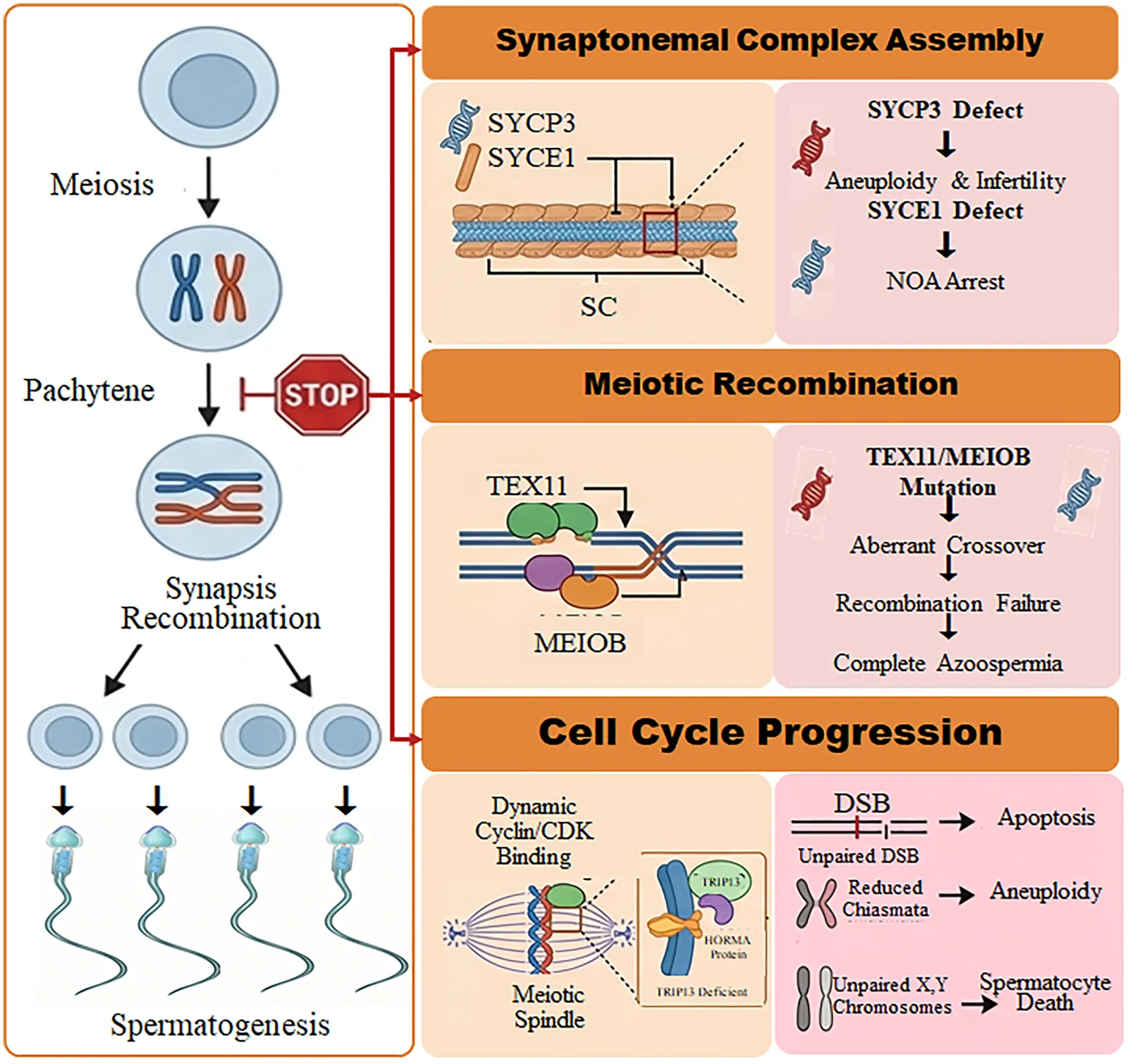
Meiotic Arrest and Spermatogenic Arrest. Meiotic progression, particularly at the pachytene stage, is critical for successful spermatogenesis. Defects in three core processes lead to meiotic arrest and infertility. *Synaptonemal Complex Assembly*: Mutations in components like SYCP3 and SYCE1 disrupt synaptonemal complex (SC) formation, causing aneuploidy, infertility, and NOA arrest. *Meiotic Recombination*: Impairment of recombination factors TEX11 and MEIOB results in aberrant crossover formation, recombination failure, and complete azoospermia. *Cell Cycle Progression:* Defects in spindle assembly, such as TRIP13 deficiency, lead to unresolved double-strand breaks (DSBs), reduced chiasmata, unpaired sex chromosomes, and subsequent spermatocyte apoptosis or aneuploidy, ultimately blocking spermatogenesis.

Screening for biallelic pathogenic variants in NOA men has implicated multiple meiotic genes, including the synaptonemal complex components (e.g., *SYCP3*, *SYCE1*), meiotic recombination factors (e.g., *TEX11*, *MEIOB*), and cell cycle regulators (Yatsenko et al., 2015; Chotiner et al., 2024). Among these, the synaptonemal complex is indispensable for homologous chromosome pairing and recombination, and its functional impairment precludes progression beyond the pachytene stage (Bolcun-Filas et al., 2009); accordingly, mutations in *SYCE1* have been shown to induce meiotic arrest (Pashaei et al., 2020). In addition, *TEX11* (testis-expressed 11) plays a central role in meiotic recombination and chromosomal synapsis; its disruption leads to complete azoospermia characterized by spermatocyte arrest (Sha et al., 2018). Notably, *TEX11* mutations represent one of the more frequent causes of meiotic arrest, accounting for approximately 1%–2% of cases in certain NOA populations (Yang et al., 2015). Furthermore, *TRIP13* functions as a critical driver of cell cycle progression in spermatocytes. Loss of *TRIP13* triggers meiotic prophase I arrest and apoptosis, largely as a consequence of unresolved DNA double-strand breaks and defective synapsis, which is manifested by persistent HORMAD proteins and asynapsed sex chromosomes. These molecular defects, particularly failed double-strand break repair and chromosome misalignment, ultimately culminate in spermatogenic failure and male infertility (Chotiner et al., 2024).

### Sertoli Cell-Specific Genes and niche defects

Apart from the germ cell-intrinsic factors, genes expressed in Sertoli cells represent another therapeutic target (Kanatsu-Shinohara et al., 2026). Sertoli cells are responsible for creating the specialized microenvironment required for germ cell development, forming the blood-testis barrier (BTB), secreting nutrients and growth factors, and providing structural support (Thumfart and Mansuy, 2023).

The critical role of Sertoli cells is confirmed by FSH receptor (*FSHR*) or androgen receptor (*AR*) gene mutations, which impair Sertoli cell responsiveness to hormonal stimulation during spermatogenesis (Abel et al., 2008) (Figure 3A). More recently, mutations in genes encoding BTB components have been found in NOA men. For example, *CLDN11* (*claudin-11*) encodes a tight junction protein that maintains BTB integrity, is critical for normal spermatogenesis in mice, and *CLDN11* variants lead to meiotic and maturation arrests during spermatogenesis (Mazaud-Guittot et al., 2010) (Figure 3B).

**FIGURE 3:**
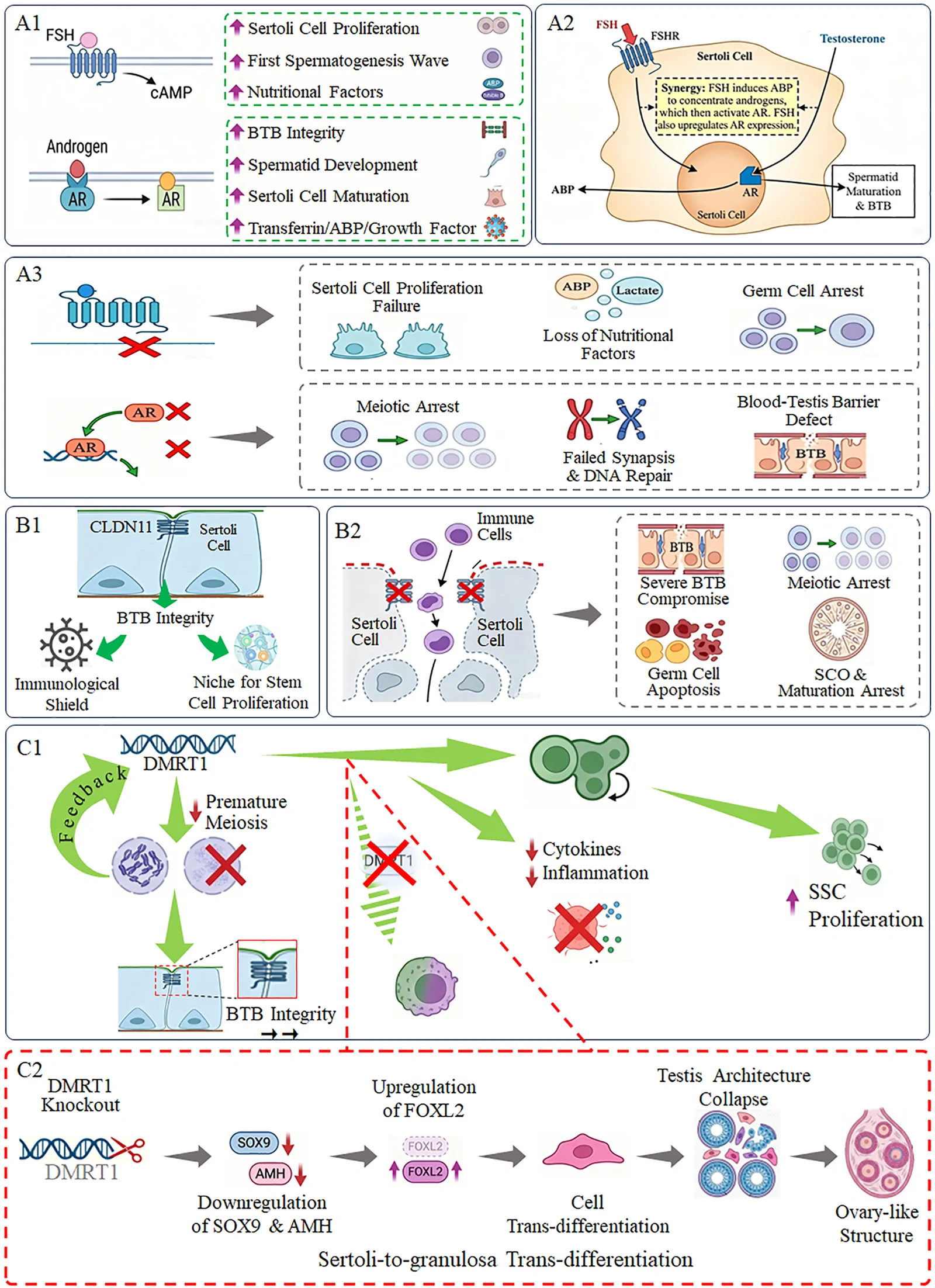
Sertoli Cell-Specific Genes and Testicular Niche for Spermatogenesis. **(A)** *FSH and androgen signaling coordinate Sertoli cell function and spermatogenesis:* FSH binds to FSHR on Sertoli cells, activating cAMP signaling to promote Sertoli cell proliferation—a prerequisite for initiating the first wave of spermatogenesis **(A1)**. Testosterone acts in parallel through the androgen receptor (AR) **(A2)**. Mechanistically, FSH induces the expression and secretion of androgen-binding protein (ABP), which concentrates androgens within the seminiferous tubule microenvironment, while also upregulating AR expression to enhance androgen sensitivity. The cooperative FSH–androgen axis regulates nutritional factors, maintains BTB integrity, and supports spermatid development, ultimately driving Sertoli cell maturation alongside the secretion of transferrin, ABP, and growth factors **(A3)**. Loss of functional FSHR and AR signaling impairs ABP and lactate secretion-reducing androgen availability and metabolic support for germ cells-thereby disrupting BTB integrity and creating a hostile microenvironment that fails to support spermatid maturation. **(B)** *CLDN11 establishes the BTB and immune-privileged niche*: CLDN11 is a tight junction protein expressed in Sertoli cells, forming paracellular sealing strands between adjacent cells that constitute the BTB **(B1)**. This barrier creates an immune-privileged microenvironment-an “immunological shield”-that protects developing germ cells, including SSCs, from autoimmune attack, while also supporting Sertoli cell proliferation and seminiferous epithelial integrity. CLDN11 deficiency disrupts BTB sealing, abolishes the immunological shield and exposes germ cells to immune destruction, impairs Sertoli cell proliferation, and compromises the stem cell niche, collectively disrupting spermatogenesis **(B2)**. **(C)** *DMRT1 maintains testicular homeostasis:* Under physiological conditions, DMRT1 suppresses premature meiosis, maintains BTB integrity in Sertoli cells, inhibits cytokine production and inflammatory responses, and promotes SSC proliferation within the germ cell lineage **(C1)**. Conversely, DMRT1 knockout reduces SOX9 and AMH expression while upregulating FOXL2, triggering Sertoli-to-granulosa trans-differentiation. These changes ultimately collapse testicular architecture and lead to the formation of an ovary-like structure **(C2)**.

The transcription factor *DMRT1* (double sex and mab-3 related transcription factor 1), expressed in both Sertoli cells and germ cells, is essential for maintaining the balance between self-renewal and differentiation of spermatogonial stem cells (SSCs) (Matson et al., 2010). *DMRT1* haploinsufficiency causes teratozoospermia and infertility in humans, while its complete loss disrupts Sertoli cell differentiation and germ cell survival (Marić et al., 2025) (Figure 3C).

Beyond these genetically defined somatic and germ cell-intrinsic defects, accumulating evidence from *in vitro* human testicular models has expanded the repertoire of therapeutic strategies for NOA. Recent advances in these model systems have propelled gene therapy exploration along two principal axes: Sertoli cell functional restoration and germ cell regeneration. Chen et al. demonstrated that overexpression of a p-FAK-Y407E phosphomimetic mutant rescues PFOS-induced Sertoli cell injury, thereby preserving the spermatogenic niche (Chen H. et al., 2017). Liu et al. further revealed that hsa-miR-100-3p regulates Sertoli cell proliferation and apoptosis via SGK3 binding, highlighting non-coding RNA modulation as an additional therapeutic avenue (Liu et al., 2021). On the germ cell front, Zhang et al. achieved direct reprogramming of human Sertoli cells into male germline stem cells with self-renewal and differentiation capacities through overexpression of DAZL, DAZ2, and BOULE, offering a potential route to autologous germ cell generation (Zhang et al., 2021). Concurrently, Feng et al. identified a novel SYCE1 exon mutation associated with NOA, providing a new target for genetic diagnosis and gene editing (Feng et al., 2022). Collectively, these studies illustrate that current NOA gene therapy research spans molecular mechanism dissection, non-coding RNA regulation, cellular reprogramming, and pathogenic gene identification. However, these findings remain largely confined to *in vitro* models, and clinical translation faces substantial challenges in delivery optimization, long-term safety, and *in vivo* validation, which are hurdles that must be prioritized in future investigations.

Against this backdrop of target discovery and pathway validation, the selection of an appropriate therapeutic modality, whether mRNA replacement, gene editing, or cell-based reprogramming, depends critically on the nature of the underlying genetic defect, the targeted cell type, and the desired duration of therapeutic effect.

### PDHA2: functions and A paradigm for mRNA replacement therapy

*PDHA2* (Pyruvate Dehydrogenase E1 Alpha 2) is a critical testis-specific gene that encodes an essential subunit of the pyruvate dehydrogenase complex. It plays an indispensable role in spermatogenesis and male fertility by providing ATP and metabolic intermediates required for germ cell development (Wang et al., 2025). Beyond the metabolic function, *PDHA2* localizes to both mitochondria and the nucleus, where it modulates chromosome structure and ensures proper crossover formation during meiosis. This dual localization underscores its unique position at the intersection of energy metabolism and epigenetic regulation during germ cell maturation (Figure 4).

**FIGURE 4:**
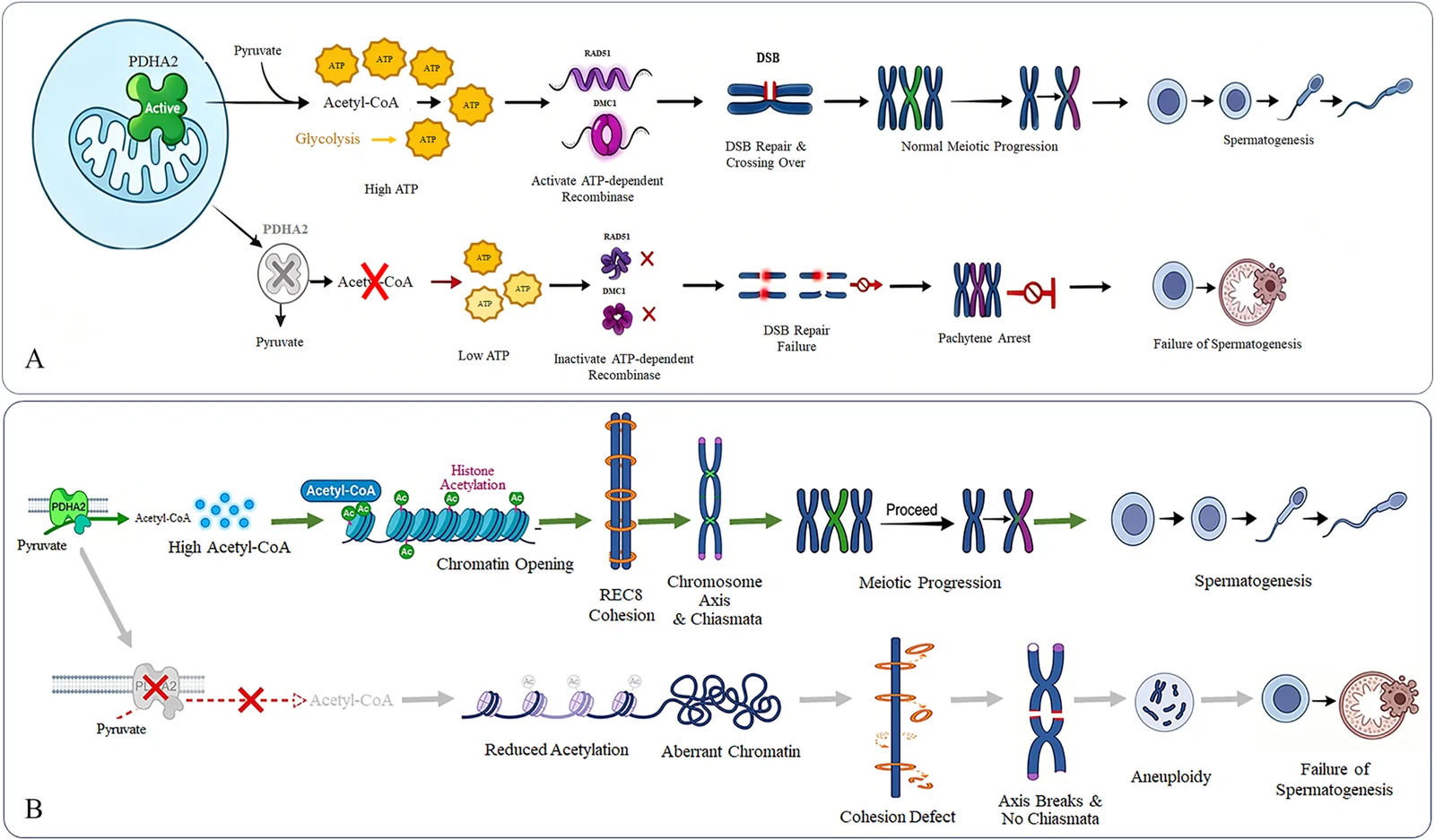
Dual Roles of PDHA2 in Spermatogenesis: Metabolic Regulation and Epigenetic Control. **(A)** *PDHA2 drives meiotic progression via ATP-dependent recombinase activity:* In wild-type cells (upper), mitochondrial PDHA2 converts pyruvate to acetyl-CoA, sustaining ATP production. Elevated ATP activates recombinases RAD51 and DMC1, enabling efficient DSB repair and homologous recombination, thus ensuring normal meiosis and mature sperm production. In PDHA2-deficient cells (lower), impaired pyruvate metabolism reduces ATP, compromising recombinase activity and causing DSB repair failure, pachytene arrest, and spermatogenic failure. **(B)** *PDHA2 regulates chromatin dynamics via acetyl-CoA-dependent histone acetylation*: In wild-type cells (upper), PDHA2 maintains high acetyl-CoA levels, promoting histone acetylation, chromatin decondensation, and REC8-mediated cohesin assembly. These events support chromosome axis formation, chiasmata development, and uninterrupted meiosis, enabling complete spermatogenesis. In PDHA2-deficient cells (lower), reduced acetyl-CoA diminishes histone acetylation, leading to aberrant chromatin compaction, cohesion defects, axis instability, and chiasmata loss, ultimately causing aneuploidy and spermatogenic failure.

The recent success of testicular mRNA delivery in a NOA mouse model positions *PDHA2* as a paradigmatic target for such an approach, given its critical, germ cell-specific role in meiosis (Mashiko et al., 2025). *PDHA2* encodes a testis-specific E1 alpha subunit of the pyruvate dehydrogenase complex, a mitochondrial enzyme that catalyzes the conversion of pyruvate to acetyl-CoA (Dahl et al., 1990). Unlike *PDHA1*, which is ubiquitously expressed in somatic cells (Johnson et al., 2001), *PDHA2* expression is restricted to male germ cells (Dahl et al., 1990). This germ cell-specific expression pattern reflects the unique metabolic requirements of meiosis, which is a highly energy-demanding process that relies heavily on pyruvate (Rong et al., 2026). Furthermore, *PDHA2* knockout mouse model recapitulates the human phenotype, with homozygous mutant males exhibiting complete meiotic arrest at the pachytene stage and absence of haploid germ cells (Pan et al., 2025) (Figure 5).

**FIGURE 5:**
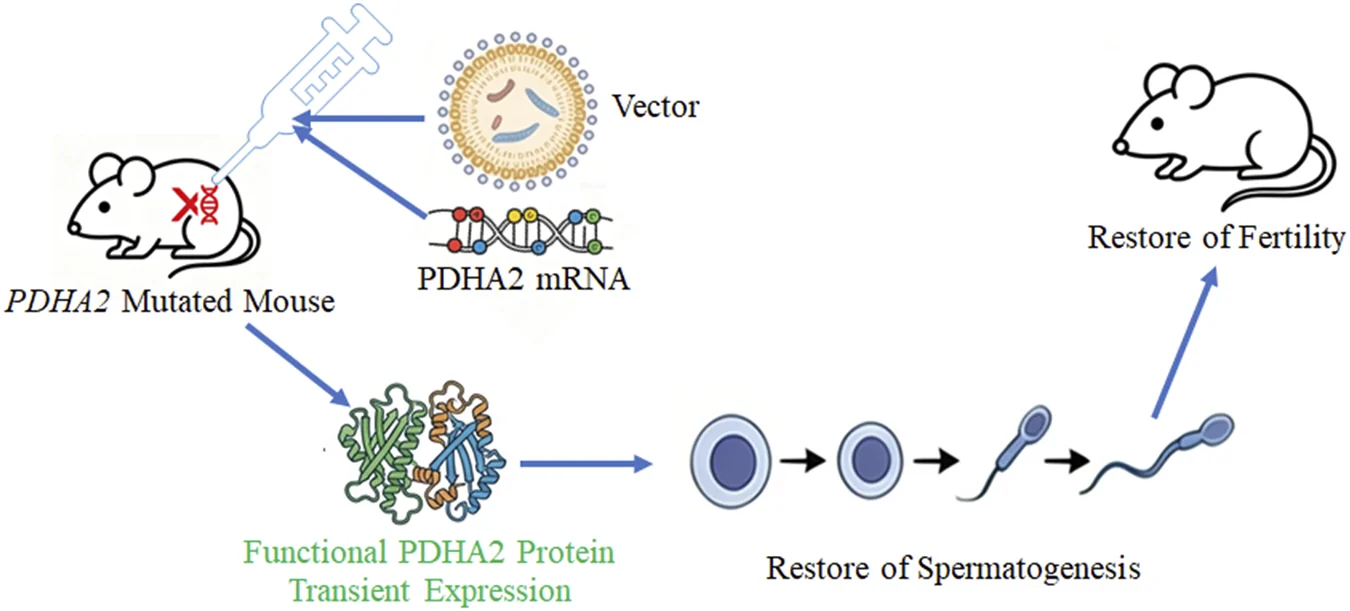
PDHA2 mRNA-mediated Rescue of Spermatogenesis and Fertility in PDHA2-deficient Mice. PDHA2 gene knockout (KO) mice harbor a mutated PDHA2 gene (red) and exhibit impaired spermatogenesis, resulting in infertility. Delivery of exogenous PDHA2 mRNA via a vector enables transient expression of functional PDHA2 protein in germ cells, which compensates for the missing enzyme during the critical pachytene-stage window of meiosis. This transient protein replacement restores meiotic progression and spermatogenesis, ultimately recovering the fertility of treated mice.

## Delivery technologies for testicular gene therapy

### Anatomical and physiological barriers of testis as a therapeutic target

Effective gene therapy for NOA must deliver genetic material to specific testicular cells, yet testicular anatomy poses a substantial obstacle (Cheng and Mruk, 2012). The testis comprises the interstitial space, which houses Leydig cells and blood vessels, and seminiferous tubules, where spermatogenesis occurs (Hammadeh et al., 2021). Within tubules, Sertoli cells establish the BTB, creating immune privilege while restricting macromolecular and vector access (Mruk and Cheng, 2015; Fijak and Meinhardt, 2006; Li et al., 2012). SSCs reside in the basal compartment outside the BTB, rendering them accessible via systemic or interstitial routes, whereas differentiating germ cells in the adluminal compartment remain protected behind the BTB, necessitating barrier disruption or direct intratubular injection (de Rooij, 2017). Sertoli cells are post-mitotic and long-lived, suitable for diverse vectors, whereas SSCs are rare and slow-cycling, posing challenges for vectors that depend on active division for integration (Damyanova et al., 2024). The following content provides a comparative analysis of various gene delivery systems, including both viral vectors and non-viral methods, alongside an introduction of the diverse routes of administration employed in gene therapy (Table 1).

**TABLE 1:** Delivery technologies for testicular gene therapy.

Delivery systems	Characteristics	Types	Key content and features	Advantages & Limitations	References
Viral Vectors	High transduction efficiency and sustained expression Different serotypes exhibit distinct tropisms for testicular cell types	Adenoviral Vectors	dsDNA, high efficiency in dividing/non-dividing cells Predominantly transduce Sertoli cells; minimal germ cell transduction Functional rescue in Sl/Sld mice via SCF delivery	Advantages: High transduction efficiency, no integration required. Limitations: Strong immunogenicity, short transgene expression, pre-existing immunity in humans, prevents redosing	(Sharpe et al., 2003; Crystal, 2014; Wang et al., 2007; Thacker et al., 2009)
		Adeno-Associated Viral Vectors	ssDNA, excellent safety profile, long-term expression in post-mitotic tissues AAV1, AAV8, AAV9 show highest promise for testicular delivery AAV9 crosses BTB to transduce both Sertoli and germ cells AAV1-mediated HITI in Cldn11-deficient mice restored BTB and spermatogenesis for ≥6 months	Advantages: Low immunogenicity, long-term expression, multiple serotypes available. Limitations: Small packaging capacity (∼4.7 kb), low-frequency integration events (insertional mutagenesis risk), potential germline transmission	(Watanabe et al., 2018; Wang et al., 2019; Mingozzi and High, 2013; Matsuzaka and Yashiro, 2024; Wu et al., 2010; Chandler et al., 2017; Kaeppel et al., 2013; Zhang et al., 2025)
		Lentiviral Vectors	Integrate into host genome, long-term expression Transduce Sertoli cells and SSCs *Ex vivo* SSC editing + transplantation produces genetically modified sperm *In vivo* delivery leads to germ cell integration and heritable modifications	Advantages: Transduce dividing and non-dividing cells, long-term stable expression. Limitations: High insertional mutagenesis risk; *in vivo* delivery leads to germline transmission (confirmed in mice); major ethical and safety concerns severely limit applicability for NOA.	(Mátrai et al., 2010; Hamra et al., 2002; Kim et al., 2019; Gurumoorthy et al., 2022; Sato et al., 2020)
Non-Viral Delivery Systems	Reduced immunogenicity, unlimited packaging capacity Easy manufacturing No genomic integration	Lipid Nanoparticles	Composed of ionizable lipids, phospholipids, cholesterol, PEG-lipids Endosomal escape via ionizable lipids Flexible cargo: mRNA, self-amplifying RNA, circular RNA. Rete testis injection of LNP-mRNA (*Pdha2*) restored spermatogenesis in NOA mouse model; ICSI produced 26 healthy offspring; whole-genome sequencing confirmed no genomic abnormalities. miRNA-mediated detargeting (miR-471 target sites) achieved germ cell-specific expression.	Advantages: Low immunogenicity, no integration risk, unlimited packaging capacity, scalable manufacturing, clear clinical translation pathway (based on mRNA vaccine precedent). Limitations: Testicular delivery efficiency requires further optimization (∼55% tubular coverage in mice); long-term safety of lipid components unknown; immunogenicity risk with repeated dosing	(Mashiko et al., 2025; Wang et al., 2023; Lummerstorfer and Lächelt, 2026; Patel et al., 2020; Kowalski et al., 2019; Hou et al., 2021; Yin et al., 2014)
		Polymer-Based Nanoparticles	Materials: PLGA, PEI, chitosan Tunable properties, sustained release, established safety profiles PLGA-GDNF delivery enhanced SSC maintenance and spermatogenesis	Advantages: Wide range of materials, tunable properties, sustained release characteristics. Limitations: Lower transfection efficiency than LNPs or viral vectors; PEI cytotoxicity at high concentrations	(Niculescu and Grumezescu, 2021; Mikušová and Mikuš, 2021; Luten et al., 2008; Pack et al., 2005; Lv et al., 2006)
		Naked mRNA Delivery	Direct testicular injection of naked *Cldn11* mRNA restored spermatogenesis in Cldn11-deficient mice Triggered innate immune response but did not impair offspring quality	Advantages: Manufacturing simplicity, no vector-related toxicity. Limitations: Rapid RNase degradation, poor cellular uptake in most tissues. Testicular immune privilege may facilitate efficacy	(Kanatsu-Shinohara et al., 2026)
Route of Administration	Systemic vs. local delivery critically determines therapeutic success	Systemic Administration	Intravenous injection: simple and minimally invasive BTB restricts paracellular diffusion; testicular endothelium limits nanoparticle access High first-pass liver clearance; minimal testicular transduction	Advantages: Simple procedure, minimally invasive. Limitations: BTB and testicular endothelium significantly limit delivery efficiency; high hepatic clearance	(Zhao et al., 2025)
		Interstitial Injection	Direct injection into testicular interstitium bypasses systemic barriers Efficient transduction of Leydig cells and peritubular myoid cells BTB prevents vector diffusion into adluminal compartment, leading to the limited germ cell access	Advantages: Bypasses systemic barriers, high transduction efficiency for interstitial cells. Limitations: BTB restricts germ cell access; requires surgical procedure	(Mruk and Cheng, 2015)
		Seminiferous Tubule Injection	Most targeted but technically challenging; impractical for whole-testis treatment Primarily used for experimental studies (lineage tracing, tropism assessment)	Advantages: Highest targeting specificity, direct delivery to seminiferous tubules. Limitations: Technically demanding, difficult to cover entire testis, limited clinical practicality	(Zhao et al., 2019)
Cell-Type Specific Targeting Strategies	Essential to minimize off-target effects and maximize efficacy	Promoter-Based Targeting	Transcriptional restriction via cell-specific promoters Sertoli cell: *WT1, SOX9, AMH* promoters Germ cell: *DAZL* promoters Used 2-kb *Cldn11* promoter fragment for Sertoli cell-specific CLDN11 expression	Advantages: Transcriptional-level regulation, high specificity. Limitations: Promoter size may limit vector packaging capacity; tissue cross-reactivity may reduce specificity	(Papadakis et al., 2004; Nicholas et al., 2009; Zhang et al., 2025)
		MicroRNA-Mediated Detargeting	Post-transcriptional suppression via miRNA target sites in 3' UTR. Inserted *Dsc1* 3' UTR (miR-471 targets) to suppress translation in Sertoli cells while permitting germ cell expression	Advantages: No need for large promoter cloning; synergistic with transcriptional control; high flexibility. Limitations: Depends on specific miRNA expression profiles; may exhibit species differences	(Mashiko et al., 2025)

### Viral vectors

Viral vectors are prized for high transduction efficiency and sustained expression, with serotype-specific tropisms for Leydig cells, Sertoli cells, or SSCs (Sharpe et al., 2003; Watanabe et al., 2018). Adenoviral vectors, which are non-enveloped, double-stranded DNA viruses, transduce dividing and non-dividing cells efficiently (Crystal, 2014), primarily targeting Sertoli cells via Coxsackievirus and adenovirus receptor (CAR) on basolateral surfaces (Wang et al., 2007), but strong immunogenicity limits expression duration and prevents redosing, with pre-existing immunity further reducing efficacy (Yao et al., 2024). Adeno-associated virus (AAV) vectors are small, non-enveloped, single-stranded DNA viruses enabling long-term expression in post-mitotic tissues with low immunogenicity (Wang et al., 2019; Mingozzi and High, 2013; Matsuzaka and Yashiro, 2024); AAV1, AAV8, and AAV9 are most promising vectors (Wang et al., 2019), and AAV9 has demonstrated the ability to transduce both Sertoli and germ cells following intratubular injection, a route that bypasses the BTB by delivering vectors directly into the seminiferous tubule lumen (Watanabe et al., 2018). However, small packaging capacity (∼4.7 kb) and low integration frequency raise concerns about insertional mutagenesis and germline transmission for AAVs (Wu et al., 2010; Chandler et al., 2017; Kaeppel et al., 2013). In addition, Lentiviral vectors offer stable genomic integration in dividing and non-dividing cells (Mátrai et al., 2010), efficiently transducing Sertoli cells and SSCs (Hamra et al., 2002; Kim et al., 2019). However, *in vivo* delivery causes both somatic and germ cell transduction, with confirmed germline transmission in mice raising major ethical and safety issues that severely limit their applicability for NOA gene therapy (Gyngell et al., 2017; Gurumoorthy et al., 2022; Sato et al., 2020).

### Non-viral delivery systems

Concerns with viral vector’s safety, such as immunogenicity, insertional mutagenesis, and germline transmission, have driven the development of non-viral systems, which offer reduced immunogenicity, unlimited packaging capacity, and transient, integration-free expression (Wang et al., 2023; Lummerstorfer and Lächelt, 2026). Lipid nanoparticles (LNPs) are the leading non-viral platform: composed of ionizable lipids, phospholipids, cholesterol, and PEG-lipids that self-assemble to encapsulate cargo, they enable transient expression, accommodate diverse nucleic acids, and are chemically synthesizable (Patel et al., 2020; Kowalski et al., 2019). Mashiko et al. used LNP-mediated mRNA delivery via rete testis injection in a Pdha2-knockout NOA mouse model, achieving widespread tubular distribution and restoring meiotic progression (Mashiko et al., 2025). By incorporating miRNA target sequences into the *Pdha2* 3' UTR, they achieved Sertoli-cell translational suppression while permitting germ cell expression (Mashiko et al., 2025). The treated mice produced 26 healthy offspring, with whole-genome sequencing confirming no abnormalities, providing definitive proof-of-concept for LNP-mRNA therapy (Mashiko et al., 2025). Polymer-based nanoparticles (PLGA, PEI, chitosan) offer tunable properties and sustained release (Niculescu and Grumezescu, 2021; Mikušová and Mikuš, 2021; Luten et al., 2008) but show lower transfection efficiency than LNPs or viral vectors, with cationic polymers such as PEI raising toxicity concerns (Pack et al., 2005; Lv et al., 2006). Naked mRNA delivery has also proven effective in the testis: injecting naked *Cldn11* mRNA into *Cldn11*-deficient mice restored spermatogenesis and yielded healthy offspring (Kanatsu-Shinohara et al., 2026). This approach is simple to manufacture but faces rapid RNA degradation; its success may reflect unique testicular microenvironment features, including the efficient uptake of mRNA by Sertoli cells (Kanatsu-Shinohara et al., 2026).

### Routes of administration

The success of gene therapy hinges critically on the administration route—systemic vascular delivery or direct testicular injection (Table 1). Systemic routes are straightforward and minimally invasive but face formidable barriers: the blood–testis barrier limits paracellular diffusion, and testicular endothelium restricts nanoparticle and viral vector entry (Zhao et al., 2025). Interstitial injection bypasses these systemic hurdles, directly accessing Leydig, peritubular, and basal Sertoli cells (Pack et al., 2005); however, the blood–testis barrier still blocks diffusion into the adluminal space, restricting transduction to exterior cells (Mruk and Cheng, 2015). Seminiferous tubule injection offers superior targeting but is technically demanding and impractical for whole-testis use, largely confined to localization-specific experiments (Zhao et al., 2019).

## Targeting strategies in testicular gene therapy: from cell type selection to expression specificity

### Germline versus somatic targeting

A fundamental consideration in designing gene therapy for NOA is whether to target germ cells directly or their supporting somatic cells (Kanatsu-Shinohara et al., 2026). Germ cell targeting aims to correct genetic defects within SSCs or their progeny, potentially restoring spermatogenesis and producing genetically normal sperm sustainably (Macedo and Alves-Lopes, 2026). However, this approach raises substantial ethical concerns, as genetic modifications in SSCs could be transmitted to future generations (Lanphier et al., 2015). Moreover, the relative quiescence of SSCs limits vector delivery efficiency (Oatley and Brinster, 2012). In contrast, somatic cell targeting which aims to rectify Sertoli cell defects that indirectly impair germ cell development, avoids the ethical issues of germline editing by not modifying germ cells genetically (Griswold, 2016; Orwig et al., 2020). Sertoli cells are often considered more practical targets than SSCs due to their greater accessibility and higher transduction efficiency with various viral and non-viral vectors (Kanatsu-Shinohara et al., 2026). However, somatic targeting is applicable only to NOA cases with somatic defects, not germ cell-autonomous abnormalities (Peng et al., 2025). In Sertoli-deficient conditions, restoring the somatic niche can indirectly support germ cell development; conversely, mutations in germ cell-intrinsic meiotic genes, such as *PDHA2*, require direct germ cell targeting (Mashiko et al., 2025).

### Cell-type specific targeting strategies

Achieving cell-type specific expression is essential for minimizing off-target effects and ensuring therapeutic efficacy. Two principal strategies have been developed for testicular applications (Table 1).

The first, promoter-based targeting employs tissue- or cell-type specific promoters to restrict expression to cells harboring the appropriate transcription factor milieu (Papadakis et al., 2004). Sertoli cell-specific promoters (e.g., *AMH*) (Lécureuil et al., 2002) and germ cell-specific promoters (e.g., *DAZL*) (Nicholas et al., 2009) have been utilized. In addition, in the homology-independent targeted integration (HITI)-mediated editing strategy, a 2-kb *Cldn11* upstream fragment containing Sertoli cell-specific cis-regulatory elements has been used to drive *CLDN11* expression recapitulating endogenous expression patterns in transgenic mice (Zhang et al., 2025).

The second strategy, microRNA-mediated detargeting operates post-transcriptionally by incorporating target sequences for miRNAs enriched in off-target cells into the 3' UTR of mRNA constructs, suppressing expression in undesirable cell types while permitting translation in target cells (Kitamura and Namekawa, 2025). Mashiko and colleagues achieved germ cell-specific expression of *Pdha2* mRNA by inserting the *Dsc1* 3' UTR containing target sites for Sertoli cell-enriched miR-471 into their mRNA construct (Mashiko et al., 2025). This approach avoids the need for large cell-type specific promoters and can synergize with promoter-based strategies (Kitamura and Namekawa, 2025).

## Preclinical proof-of-concept: gene therapy successes in NOA animal models

The feasibility of gene therapy for NOA has been firmly established through two landmark proof-of-concept studies in genetically defined mouse models, demonstrating that both germ cell-targeted mRNA replacement and somatic cell gene editing can restore spermatogenesis and produce viable offspring.

### Germ cell-targeted mRNA replacement: the Pdha2 paradigm

Mashiko and colleagues achieved the first definitive demonstration of gene therapy-mediated restoration of fertility in a NOA mouse model through LNP-mediated mRNA delivery targeting the *Pdha2* gene (Mashiko et al., 2025). PDHA2 is a testis-specific pyruvate dehydrogenase subunit essential for meiotic progression; its deficiency causes pachytene-stage arrest and complete azoospermia (Pan et al., 2025).

This study incorporated several innovative design features, including an optimized LNP formulation that achieved approximately 55% tubular delivery after rete testis injection, incorporation of miR-471 target sites in the *Dsc1* 3' UTR to enable Sertoli cell-specific translational suppression while preserving germ cell expression, and use of m1Ψ-modified mRNA to reduce immunogenicity. Treated males produced sperm that yielded 26 healthy, fertile offspring via ICSI, and whole-genome sequencing confirmed no genomic aberrations. The roughly 5-day expression window proved sufficient to restore spermatogenesis, indicating that even transient protein replacement during this critical period can achieve therapeutic efficacy (Mashiko et al., 2025).

### Somatic cell gene editing: HITI-mediated rescue of Cldn11 deficiency

Complementing the germ cell-targeted approach, Zhang and colleagues employed HITI to correct Sertoli cell defects in *Cldn11*-deficient mice (Zhang et al., 2025). As described above, *Cldn11* deficiency disrupts BTB integrity and causes meiotic arrest (Mazaud-Guittot et al., 2010). Using AAV1 vectors delivered via testicular tubular injection, the authors delivered CRISPR/Cas9 components and a Sertoli cell-specific donor template encoding *CLDN11* for integration into the endogenous locus. Following treatment, *CLDN11* expression was restored, BTB integrity was re-established, and spermatogenesis resumed, with treated mice producing fertile offspring. Therapeutic effects persisted for at least 6 months (Zhang et al., 2025).

Collectively, these studies establish several key principles for testicular gene therapy, demonstrating that both germ cell-intrinsic and somatic cell defects can be therapeutically targeted, that appropriately timed transient mRNA delivery can rescue meiotic arrest, that gene editing in post-mitotic Sertoli cells can achieve durable effects, and that the HITI strategy enables efficient knock-in within non-dividing cells without homologous recombination. At the same time, these proof-of-concept findings reveal critical translational hurdles requiring resolution prior to clinical use, such as optimizing delivery for the larger human testis, validating long-term safety, and developing scalable manufacturing processes.

### Rationale for mRNA over DNA-based approaches

mRNA-based therapeutics have emerged as a compelling alternative to DNA-based gene therapy for numerous applications, including the treatment of NOA (Mashiko et al., 2025; Kitamura and Namekawa, 2025), largely attributable to its several features for testicular applications (Figure 6; Table 2).

**FIGURE 6:**
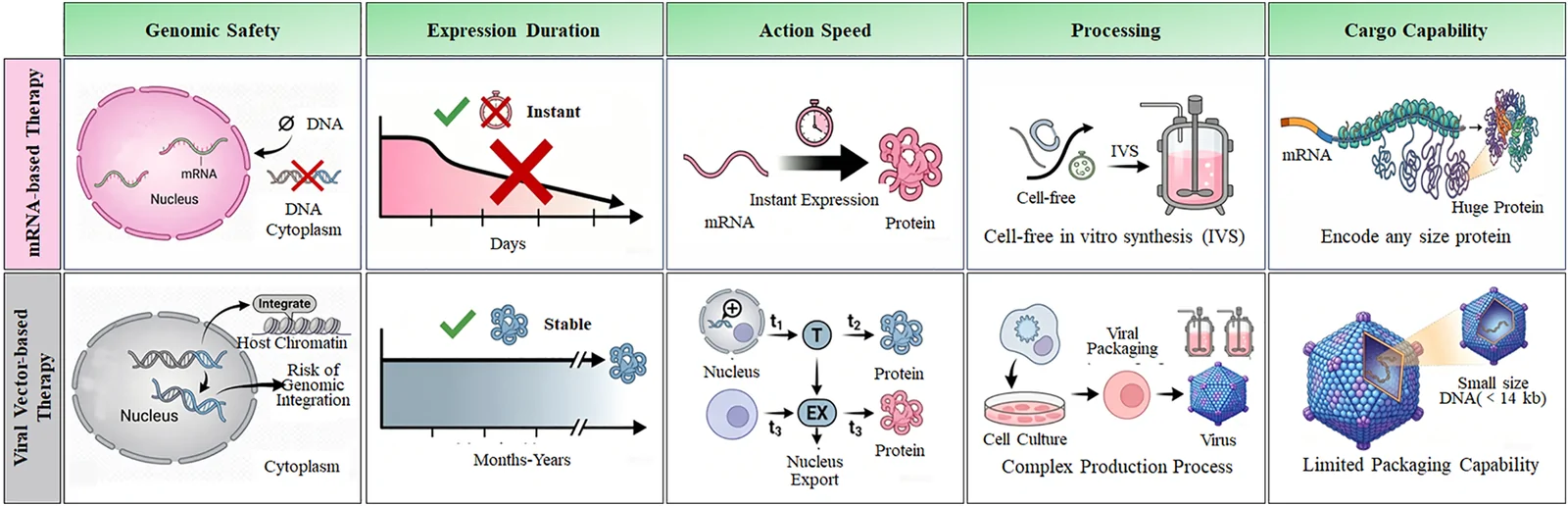
Comparative Overview of mRNA-based and Viral Vector-based Therapies. This figure compares mRNA-based and viral vector-based therapeutic approaches across four critical parameters. *Genomic safety:* mRNA remains in the cytoplasm and does not integrate into the host genome, whereas viral vectors carry a risk of genomic integration via insertion into host chromatin. *Expression duration:* mRNA mediates transient expression (cytoplasmic, no DNA intermediate), while viral vector-based delivery achieves long-lasting expression (months to years) due to nuclear residence and potential integration. *Action speed:* mRNA enables instant protein expression upon cytoplasmic delivery, whereas viral vectors require nuclear trafficking and transcriptional activation, resulting in slower onset over months to years. *Processing and cargo capability:* mRNA is produced cell-free via *in vitro* synthesis (IVS), can encode proteins of any size, and involves no packaging constraints. In contrast, viral vectors rely on cell culture and complex production processes, with limited packaging capacity (typically <14 kb for small DNA cargoes).

**TABLE 2:** Advantages of mRNA therapy for NOA treatment.

Advantage	Core feature	Relevance and significance	Comparative advantage over DNA/Viral vectors	References
Genomic Safety	Acts in the cytoplasm without nuclear entry, completely eliminating risks of genomic integration and insertional mutagenesis	Critically important for testicular gene therapy to prevent unintended transmission of genetic modifications to offspring via germ cells	Superior to: DNA vectors (integration risk) and viral vectors (insertional mutagenesis, germline transmission risk)	(K et al., 2008)
Controllable Expression	Inherently transient expression, with protein production lasting days to weeks depending on mRNA stability and cell division rates	Aligns well with treating meiotic arrest, where protein replacement during a defined developmental window may suffice to restore spermatogenesis without permanent genome alteration	Superior to: Viral vectors (long-term, uncontrolled expression) and DNA vectors (persistent expression). Limitation: May require repeated dosing	(Mashiko et al., 2025; Kitamura and Namekawa, 2025; K et al., 2008)
High Efficacy	Does not require nuclear transcription, enabling rapid onset of protein expression even in post-mitotic cells	Offers a faster therapeutic effect compared to DNA-based approaches by bypassing nuclear transport and complex transcription processes	Superior to: DNA vectors (require nuclear transcription, splicing, export) and viral vectors (require nuclear delivery)	(Rosa et al., 2021)
Manufacturing Accessibility	Cell-free, scalable manufacturing process that is relatively simple compared to viral vector production	Facilitates clinical translation by leveraging the robust manufacturing platforms and regulatory pathways already established by mRNA vaccines	Superior to: Viral vectors (require cell culture, complex production, higher cost)	(Kumar et al., 2025)
Cargo Flexibility	Can encode proteins of various sizes without the same packaging constraints as viral vectors	Essential for NOA treatment, as therapeutic proteins may be large or require the inclusion of complex regulatory elements	Superior to: AAV vectors (∼4.7 kb capacity limit) and adenoviral vectors (∼8–10 kb capacity limit)	(Mashiko et al., 2025; Zhou et al., 2026; Chen et al., 2025a)

First, mRNA acts in cytoplasm without nuclear entry, eliminating risks of genomic integration and insertional mutagenesis, which is a critical consideration in the testis, where integration into germ cells could lead to unintended heritable modifications (K et al., 2008). Second, mRNA expression is inherently transient, with protein production lasting days to weeks depending on stability and cell division rates, which aligns well with the treatment of meiotic arrest, where protein replacement during a defined developmental window may suffice to restore spermatogenesis (Mashiko et al., 2025; Kitamura and Namekawa, 2025; K et al., 2008). Third, mRNA enables rapid protein expression in post-mitotic cells without requiring nuclear transcription, splicing, or export, contrasting with DNA-based approaches that involve these slower steps (Chanda et al., 2021). Fourth, mRNA manufacturing is cell-free, scalable, and simpler than viral vector production, with robust platforms and regulatory pathways established by successful mRNA vaccines that can be adapted for therapeutic use (Rosa et al., 2021; Kumar et al., 2025). Fifth, mRNA can encode proteins of any size without the packaging constraints of viral vectors, a flexibility essential for NOA where therapeutic proteins may be large or require complex regulatory elements (Mashiko et al., 2025; Zhou et al., 2026; Chen Q. et al., 2025).

### Chemical modifications for enhanced stability and reduced immunogenicity

Unmodified mRNA is inherently immunogenic and unstable, limiting its therapeutic utility (Karikó et al., 2005). Recognition of exogenous RNA by pattern recognition receptors, particularly Toll-like receptors (TLRs) 3, 7, and 8, and retinoic acid-inducible gene I (*RIG-*I), triggers innate immune responses that suppress translation and cause inflammation (Luan et al., 2024; Hornung et al., 2006).

The development of modified nucleosides has revolutionized mRNA therapeutics by enabling evasion of innate immune sensing (Andries et al., 2015). m1Ψ-modified mRNA, used in the Pfizer-BioNTech and Moderna COVID-19 vaccines, exhibits markedly reduced immunogenicity and increased protein production compared to unmodified mRNA (Nance and Meier, 2021; Yang B. et al., 2025).

Additional modifications enhance stability and translational efficiency. The 5' cap, which is a reverse 7-methylguanosine linked via a 5'-5' triphosphate bridge, protects mRNA from exonuclease degradation and recruits translation initiation factors (Furuichi and Shatkin, 2000; Cowling, 2009). Optimized cap analogs, such as CleanCap or ARCA (anti-reverse cap analog), ensure proper orientation and enhance translation (Kang et al., 2023).

The 5' and 3' untranslated regions (UTRs) influence mRNA stability and translation through recruitment of RNA-binding proteins and microRNAs; incorporation of UTRs from highly expressed genes, such as human α- or β-globin, enhances mRNA performance (Ryczek et al., 2023; Castillo-Hair et al., 2024). The poly(A) tail, typically 100–150 nucleotides in length, promotes translation initiation and protects against deadenylation (Fernandez and Mendez, 2025).

Mashiko and colleagues employed m1Ψ-modified mRNA in their LNP formulation for *Pdha2* delivery, achieving robust protein expression with minimal immunotoxicity (Mashiko et al., 2025). This modification strategy, now standard in the field, enabled therapeutic efficacy without compromising safety.

### LNP-mediated Pdha2 mRNA delivery: a paradigm shift

The successful restoration of spermatogenesis in *Pdha2* knockout mice via LNP-mediated mRNA delivery marks a paradigm shift in genetic NOA treatment (Mashiko et al., 2025). This achievement demonstrates that even complete meiotic arrest due to critical enzyme loss can be reversed by transient protein replacement within a defined developmental window. Several study features warrant detailed consideration (Table 3).

**TABLE 3:** Design strategies and key outcomes of Pdha2 mRNA therapy.

Design dimension	Strategy and features	Key outcomes and significance	Methodological limitations/Unresolved questions	References
Target Selection	Targeted the *Pdha2* gene; its deficiency causes meiotic arrest at the pachytene stage, precisely when the enzyme is normally expressed at high levels	Providing *Pdha2* mRNA during this critical developmental window enabled completion of meiosis and production of haploid germ cells	Limitation: Targets only a single gene defect; applicability to more complex NOA etiologies unknown	(Mashiko et al., 2025)
Delivery System	Optimized LNP formulation for testicular delivery, incorporating ionizable lipids, phospholipids, cholesterol, and PEG-lipids	Achieved efficient delivery to approximately 55% of seminiferous tubules following rete testis injection	Limitation: 55% tubular coverage is effective in mice, but whether this translates to clinically meaningful levels in the larger human testis is unknown; long-term safety of LNP components requires further validation	(Mashiko et al., 2025; Kitamura and Namekawa, 2025)
Targeting Specificity	Incorporated the *Dsc1* 3' UTR containing miR-471 target sites for microRNA-mediated detargeting	Suppressed expression in Sertoli cells while permitting robust translation in germ cells, thereby minimizing off-target effects	Limitation: miRNA expression profiles differ across species; efficacy in humans requires validation	(Mashiko et al., 2025)
Expression Duration	Achieved transient protein expression lasting approximately 5 days	Demonstrated that transient expression during a critical developmental window is sufficient to rescue spermatogenesis, without the need for continuous protein replacement	Limitation: Mouse spermatogenic cycle is ∼35 days versus ∼74 days in humans; a 5-day expression window may be insufficient in humans, potentially requiring repeated dosing or self-amplifying RNA platforms	(Mashiko et al., 2025)
Functional Outcomes	Evaluated sperm function post-treatment and the health of resulting offspring	Treated mice produced sperm that, via ICSI, generated healthy offspring with no evidence of genomic abnormalities, confirming that transient mRNA replacement does not compromise subsequent germline function	Limitation: Limited offspring number (n = 26); WGS only detected large-scale abnormalities (>1 Mb), cannot exclude small mutations or epigenetic changes; no multi-generational monitoring performed	(Mashiko et al., 2025)

First, *PDHA2* deficiency causes pachytene-stage arrest, during which the enzyme is normally highly expressed, and *Pdha2* mRNA delivery within this window enabled meiotic completion (Mashiko et al., 2025). Second, the LNP formulation was optimized for testicular delivery using ionizable lipids, phospholipids, cholesterol, and PEG-lipids, achieving ∼55% tubular delivery following rete testis injection (Mashiko et al., 2025; Hou et al., 2021). Third, incorporation of miRNA target sequences (miR-471 sites in the *Dsc1* 3' UTR) conferred cell-type specificity, suppressing Sertoli cell expression while allowing germ cell translation (Mashiko et al., 2025). Fourth, the ∼5-day expression window sufficed to rescue spermatogenesis, suggesting transient rather than continuous replacement is required (Mashiko et al., 2025). Fifth, functional outcomes were definitive: treated mice produced sperm that generated healthy, fertile offspring via ICSI with no genomic abnormalities, confirming that transient mRNA replacement does not impair germline function (Mashiko et al., 2025).

While the study by Mashiko et al. provides a compelling proof-of-concept, several methodological and translational limitations warrant consideration. The experiments were conducted in a mouse model with a defined genetic background and under controlled conditions, which may not recapitulate the heterogeneity of human NOA. The long-term safety of repeated LNP administrations, potential chronic inflammation from the lipid components, and the need to translate the 55% tubular delivery efficiency to a clinically meaningful level in the larger human testis remain significant challenges. Furthermore, the dependence on rete testis injection, a procedure that is feasible in mice but technically challenging in humans, underscores the urgent need for less invasive and more clinically practical delivery routes.

### Sertoli cell mRNA therapy for niche defects

Complementing the germ cell-targeted approach, Kanatsu-Shinohara and colleagues demonstrated that mRNA delivery to Sertoli cells can rescue NOA caused by somatic cell defects using *Cldn11*-deficient mice, which represent a well-established model of BTB disruption (Kanatsu-Shinohara et al., 2026; Mazaud-Guittot et al., 2010). Treatment with naked *Cldn11* mRNA restored claudin-11 expression in Sertoli cells, enabling BTB assembly and resumption of spermatogenesis (Kanatsu-Shinohara et al., 2026).

Notably, this study used naked mRNA without any delivery vehicle, relying on the intrinsic ability of Sertoli cells to take up extracellular RNA, which is a process facilitated by the testicular microenvironment despite the generally low efficiency of naked mRNA uptake (Kanatsu-Shinohara et al., 2026). Although the authors documented an innate immune response characterized by upregulation of interferon-stimulated genes, spermatogenesis proceeded normally and offspring exhibited normal imprinting patterns, suggesting that transient inflammation may be tolerable in the context of testicular mRNA therapy, though long-term consequences warrant further investigation (Kanatsu-Shinohara et al., 2026). The study’s reliance on naked mRNA uptake by Sertoli cells, while successful, raises questions about the generalizability of this approach to other cell types or genetic defects. Additionally, the potential for repeated dosing to trigger a more robust immune response or to disrupt the delicate testicular immune privilege remains unexplored. These limitations highlight the need for more comprehensive safety studies, particularly in large animal models with immune systems more similar to humans.

### Duration of expression and dosing frequency

The optimal duration of transgene expression for NOA therapy remains unclear and likely varies by genetic defect and cell type (Cannarella et al., 2023).

For meiotic arrest caused by germ cell-intrinsic defects, transient expression during the meiotic window may suffice, as demonstrated in the *Pdha2* model (Mashiko et al., 2025). In this case, protein replacement during the pachytene stage enabled the completion of meiosis, after which continued expression is unnecessary. The resulting haploid germ cells complete spermiogenesis without requiring the ongoing production of therapeutic proteins. However, longer-term expression may be required for Sertoli cell defects, because long-lived Sertoli cells are necessary to continuously maintain the BTB and support germ cell development through multiple cycles of spermatogenesis. Transient mRNA delivery restored BTB integrity and initiated long-term spermatogenesis, as spermatogenesis persisted for at least 2 months after a single injection, suggesting that transient expression of a tight-junction protein is sufficient for sustained recovery (Kanatsu-Shinohara et al., 2026). Strategies for achieving sustained expression, such as repeated dosing or use of self-amplifying RNA, may be necessary for such applications (Cao et al., 2025).

The required frequency of dosing also depends on the kinetics of spermatogenesis. In humans, the complete spermatogenic cycle lasts approximately 74 days, with meiotic progression from spermatogonial differentiation to spermiation taking approximately 64 days (Babakhanzadeh et al., 2020). A single dose providing transient expression during a critical developmental window may not align with this protracted time course. This necessitates a careful re-evaluation of dosing frequency and the potential need for sustained or pulsatile delivery strategies to synchronize with the extended human spermatogenic cycle.

## Gene editing strategies for NOA

### Principles and testicular applications of CRISPR/Cas9

CRISPR/Cas9 system, which comprises a Cas9 nuclease for creating DSBs in DNA and a single guide RNA (sgRNA) for directing Cas9 to specific genomic loci through complementary base pairing (Jiang and Doudna, 2017), has revolutionized gene editing, enabling precise modification of genomic sequences in living cells (Park et al., 2025).

CRISPR/Cas9 offers the potential for permanently correcting the genetic defects of NOA, potentially restoring endogenous gene regulation and avoiding the need for repeated dosing (Kumar, 2025). Nevertheless, the application to the testis still presents several unique challenges, such as delivery, cell-type targeting, and safety with respect to germline modification (Sikiru et al., 2025).

### Emerging precision editing tools

Beyond the canonical CRISPR/Cas9 nuclease, novel gene editing technologies are rapidly evolving and hold significant promise for NOA therapy. Prime editing and base editing enable precise single-base substitutions or small insertions/deletions without introducing a double-strand break (DSB) (Anzalone et al., 2019). By avoiding DSBs, these technologies dramatically reduce the risks of p53 pathway activation, large chromosomal deletions, and complex genomic rearrangements (Newby and Liu, 2021), which are particularly concerning when editing germline-adjacent or post-mitotic cells. Furthermore, established platforms like TALENs and ZFNs offer alternative approaches with potentially lower off-target effects, albeit with more complex protein engineering and delivery requirements (Newby and Liu, 2021; Zubair et al., 2025). The choice of editing tool will ultimately depend on the specific mutation to be corrected, the target cell type, and the desired balance between efficiency, precision, and safety.

### *Ex vivo* editing of spermatogonial stem cells

*Ex vivo* gene editing of SSCs offers a promising strategy for precise, permanent correction of male germline mutations, enabling transmission of corrected alleles to offspring and elimination of hereditary diseases (Yamada et al., 2016). In this approach, SSCs are harvested from testicular tissue, edited *in vitro* using CRISPR/Cas9, and transplanted back into the testis to colonize the niche and generate corrected sperm (Sato et al., 2015).

This strategy has been successfully demonstrated in mice, and Wu and colleagues corrected a *Crygc* mutation in SSCs, achieving restored spermatogenesis and production of healthy offspring after transplantation (Wu et al., 2015).

However, human translation faces substantial obstacles. First, efficient harvest and expansion of human SSCs *in vitro* remains challenging, as they proliferate slowly and tend to lose stem cell properties over time (Sadeghian-Nodoushan et al., 2025). Second, autologous SSC transplantation requires disruption of endogenous germ cells, typically via chemotherapy or radiation, carrying significant toxicity (Ganguli et al., 2016). Third, colonization efficiency is low, necessitating large cell numbers for meaningful spermatogenic restoration (Ishii et al., 2014).

Importantly, off-target mutations can be identified by deep sequencing, and clones with undesirable edits can be excluded, thereby providing a level of safety assurance unattainable with *in vivo* approaches (Li et al., 2020).

### *In vivo* gene editing: HITI and other strategies

As an alternative to *ex vivo* approaches, *in vivo* gene editing aims to correct genetic defects directly within the testis. This strategy circumvents the need for SSC harvest, *ex vivo* culture, and autologous transplantation, offering a more direct and potentially less technically demanding therapeutic route. However, it comes with distinct challenges, including the delivery of editing machinery to specific cell types *in vivo*, an increased risk of unintended germline editing, and the difficulty of controlling the extent and duration of editing. The following section discusses recent progress in in vivo editing strategies, with a focus on HITI in Sertoli cells.

Recently, Zhang and colleagues employed HITI to achieve *in vivo* editing in Sertoli cells of *Cldn11*-deficient mice (Zhang et al., 2025). HITI leverages non-homologous end joining (NHEJ)-mediated repair for targeted integration without requiring homologous recombination, making it particularly suitable for post-mitotic cells such as Sertoli cells (Suzuki et al., 2018). Using AAV1 vectors delivered via testicular tubular injection, the authors delivered CRISPR/Cas9 components and a Sertoli cell-specific donor template encoding *CLDN11* for integration into the endogenous locus (Zhang et al., 2025). Sperm DNA sequencing in the treated mice revealed no evidence of off-target editing or germline transmission of the integrated construct in the limited number of offspring analyzed (Zhang et al., 2025). However, as a preliminary report, these findings warrant cautious interpretation. The HITI strategy relies on NHEJ-mediated repair, which inherently predisposes to unpredictable indels, chromosomal translocations, and large structural variants at off-target loci that may evade detection by standard sequencing. Moreover, intra-testicular AAV delivery carries the risk of vector leakage into the interstitium and unintended exposure to adjacent SSCs, raising theoretical concerns regarding inadvertent germline editing even when targeting somatic Sertoli cells. Therefore, although the current data are encouraging, *in vivo* somatic cell editing for NOA cannot yet be deemed free of germline modification risk. Rigorous safety validation in large animal models, complemented by comprehensive off-target profiling using orthogonal detection methods, remains an indispensable prerequisite for clinical translation.

## Alternative and emerging therapeutic approaches

Beyond the gene therapy modalities discussed above, several alternative and complementary strategies are under active investigation for NOA. Table 4 provides a comparative overview of these emerging approaches, including SSC transplantation, *in vitro* spermatogenesis, artificial gametes from pluripotent stem cells, small molecule modulators, and combination regimens, with a critical assessment of their current status, major challenges, and translational potential. Notably, these strategies span a wide range of technology readiness levels (TRL 2–4), with SSC transplantation being the most clinically proximate, while artificial gamete generation and complete *in vitro* spermatogenesis remain long-term objectives requiring substantial foundational advances. Unlike gene therapy approaches that aim for permanent genetic correction, these alternatives offer distinct advantages such as reversibility (small molecules), applicability to patients lacking endogenous germ cells (artificial gametes), or the potential for synergistic combination with gene editing (SSC transplantation combined with *ex vivo* correction). However, each faces unique translational hurdles that must be systematically addressed before clinical integration.

**TABLE 4:** Emerging therapeutic strategies for NOA.

Strategy	Core principle	Current status & Key findings	Major challenges & Limitations	Translational potential assessment	References
SSC Transplantation	Transplantation of SSCs to colonize the niche and regenerate spermatogenesis	Results in animal models have been achieved, and this provides fertility options for prepubertal boys through SSC cryopreservation	Transplanted SSCs in cell-autonomous defects retain the same mutation, necessitating ex vivo gene editing Moreover, human SSC expansion remains challenging, and post-transplantation colonization efficiency is low	TRL 3-4 (proof-of-concept in animal models; limited human data). Near-term application: Fertility preservation in prepubertal boys. Long-term: Combination with gene editing	(Ogawa et al., 2005; van Maaren et al., 2023; Yang et al., 2025b; Mulder et al., 2016)
*In Vitro* Spermatogenesis	Complete differentiation of male germ cells from SSCs to spermatozoa in culture	Murine organ culture has succeeded, but human studies remain limited to partial spermatogenesis, with progression only to spermatocytes or early spermatids	Human spermatogenesis is prolonged (∼74 days) and elaborate. Technical difficulties in maintaining tissue viability and hormonal support, and in replicating the 3D seminiferous epithelium architecture	TRL 2-3 (murine success; human partial success only). Timeline: Long-term objective; significant technical breakthroughs required	(Nguyen et al., 2024; Galdon et al., 2016; Ibtisham and Honaramooz, 2020; Sato et al., 2011; Song and Wilkinson, 2012; de Michele et al., 2018; Medrano et al., 2018; Portela et al., 2019)
Artificial Gametes & Stem Cell-Derived Germ Cells	Generation of gametes from pluripotent stem cells (ESCs or iPSCs)	Murine studies have shown that ESCs/iPSCs can differentiate into PGCLCs and generate fertile sperm, offering a potential avenue for patients with Sertoli cell-only syndrome	Human translation remains remote, hampered by complex signaling and epigenetic reprogramming, ethical concerns over embryo use and reproductive applications, and the high safety threshold necessitated by risks of epigenetic abnormalities and tumorigenicity	TRL 2-3 (murine proof-of-concept; human translation distant). Ethical concerns: High. Timeline: Very long-term	(Hendriks et al., 2015; Murase et al., 2024; Hayashi et al., 2011; Ishikura et al., 2016; Irie et al., 2015; Cohen et al., 2017; Fang et al., 2018; Ben-David and Benvenisty, 2011)
Small Molecule Therapies	Modulation of genetic pathways using drugs to compensate for the underlying abnormality	RANKL represents a promising therapeutic target for NOA	The complexity of spermatogenesis and limited testicular drug accessibility pose major constraints. This represents a precision medicine paradigm centered on patient selection, not genetic correction	TRL 3-4 (preclinical data; early clinical signals). Advantage: Reversible, off-the-shelf, less costly than gene therapy. Limitation: Requires ongoing administration	(Blomberg Jensen et al., 2021; Andreassen et al., 2024)
Combination Strategies	Integration of multiple therapeutic approaches for synergistic effects	Gene therapy with hormonal optimization and Sertoli cell gene editing combined with autologous SSC transplantation represent potential strategies	This necessitates careful optimization and should be tailored to individual patient attributes, such as genetic diagnosis, histology, and hormonal status	TRL 2-3 (conceptual; preclinical validation limited). Potential: Highest likelihood of clinical success by addressing multiple facets of spermatogenic failure	(Sikiru et al., 2025; Mashiko et al., 2025; Hubbard et al., 2025)

SSC transplantation offers an alternative cell-based strategy for restoring fertility in NOA. In this approach, SSCs harvested from testicular tissue are expanded in culture and reintroduced into the testis to colonize the niche and regenerate spermatogenesis (Ogawa et al., 2005). For NOA resulting from SSC depletion or dysfunction, autologous transplantation could theoretically restore fertility without addressing the underlying genetic lesion; however, in cases with cell-autonomous defects, transplanted SSCs would carry the same mutation and fail to produce normal sperm (Macedo and Alves-Lopes, 2026). Although SSC transplantation combined with *ex vivo* gene editing has corrected genetic defects in animal models, human translation remains hampered by difficulties in expanding human SSCs and achieving efficient colonization post-transplantation (van Maaren et al., 2023). For prepubertal boys undergoing gonadotoxic therapy, SSC cryopreservation offers fertility preservation that could later be paired with gene editing if inherited mutations are present, which represents one of the most promising near-term applications of SSC-based approaches (Yang F. et al., 2025).

In parallel with SSC-based strategies, *in vitro* spermatogenesis could revolutionize NOA treatment by enabling sperm production independent of functional testicular tissue (Nguyen et al., 2024; Galdon et al., 2016). Despite decades of research, complete *in vitro* spermatogenesis has been achieved only in mice, with limited efficiency, initially through organ culture of testicular tissue and subsequently refined by gas-liquid interface culture (Ibtisham and Honaramooz, 2020; Sato et al., 2011). Human translation, however, faces substantial hurdles: human spermatogenesis is considerably longer (∼74 days) and more complex than the murine process, requiring technically demanding maintenance of tissue viability and hormonal support (Song and Wilkinson, 2012); moreover, the three-dimensional architecture of the human seminiferous epithelium is difficult to recapitulate *in vitro* (de Michele et al., 2018). Nevertheless, partial human spermatogenesis up to the spermatocyte or early spermatid stage has been reported, though complete differentiation remains a long-term objective (Medrano et al., 2018; Portela et al., 2019).

Even more ambitious is the generation of artificial gametes from pluripotent stem cells—embryonic stem cells (ESCs) or induced pluripotent stem cells (iPSCs)—offering an unlimited gamete source for patients lacking endogenous germ cells entirely (Hendriks et al., 2015; Murase et al., 2024). In mice, ESCs and iPSCs have been differentiated into primordial germ cell-like cells (PGCLCs) that, upon transplantation into neonatal testes, underwent spermatogenesis and produced fertile sperm (Hayashi et al., 2011); subsequent refinements achieved *in vitro* differentiation of PGCLCs into spermatids capable of generating offspring (Ishikura et al., 2016). Human translation, however, remains challenging: germ cell specification involves complex, incompletely understood signaling and epigenetic reprogramming (Irie et al., 2015), and ethical concerns regarding embryo use and gamete creation add further complexity (Cohen et al., 2017). For NOA patients with complete germ cell aplasia, such as Sertoli cell-only syndrome, autologous iPSC-derived gametes could theoretically provide patient-specific germ cells for transplantation or fertilization (Fang et al., 2018). Nevertheless, extensive safety validation is required before clinical application, given the risks of epigenetic abnormalities and tumorigenicity associated with pluripotent stem cells (Ben-David and Benvenisty, 2011).

Although not strictly gene therapy, small molecule approaches offer a promising, reversible, and precise means of treating NOA by modulating key regulators of spermatogenesis (Mo et al., 2023). For patients with specific genetic defects, these molecules could bias DNA repair toward the precise homology-directed repair (HDR) pathway over error-prone NHEJ (Li et al., 2017); however, the complexity of spermatogenesis and limited testicular accessibility have constrained their development. One notable example is the RANKL inhibitor denosumab, widely used for osteoporosis; preclinical data implicating RANKL in germ cell apoptosis and differentiation have raised interest in its application for NOA, representing one of the first precision medicine strategies based on patient selection rather than genetic correction (Blomberg Jensen et al., 2021; Andreassen et al., 2024).

Finally, the future of NOA therapy likely lies in combination regimens that integrate multiple approaches to address spermatogenic failure, with treatment choice guided by genetic diagnosis, testicular histology, and hormonal profile, thereby embodying the precision medicine paradigm and maximizing success (Hubbard et al., 2025). For patients with defined defects, gene therapy could be combined with hormonal optimization to enhance efficacy (Aydos et al., 2023); mRNA replacement, for example, may be most effective following hormonal stimulation that synchronizes germ cell development (Mashiko et al., 2025). Similarly, Sertoli cell gene editing could be paired with autologous SSC transplantation to address both niche and germ cell defects, which represents a synergistic approach with significant promise once optimized (Sikiru et al., 2025).

## Translational roadmap and regulatory pathways for clinical application

Clinical translation of gene therapy for NOA from preclinical proof-of-concept to first-in-human trials requires rigorous validation in appropriate animal models, coupled with precise genetic diagnosis to identify patients most likely to benefit from specific interventions (Sikiru et al., 2025). However, a critical consideration in this roadmap is the substantial biological differences between murine and human spermatogenesis, including disparities in cycle duration (∼35 days in mice versus ∼74 days in humans), testicular size and architecture, blood-testis barrier tightness, and the complexity of hormonal regulation. These differences suggest that delivery efficiency and the required duration of transgene expression observed in mice may not directly translate to humans, necessitating careful optimization in large animal models and, ultimately, in clinical trials.

### Regulatory pathways for first-in-human trials

The regulatory requirements for gene therapy products vary substantially depending on the modality (viral vectors, non-viral LNPs, or cell-based products). In the United States, gene therapy products are regulated by the Center for Biologics Evaluation and Research (CBER) of the Food and Drug Administration (FDA) under the framework for cellular and gene therapy products (U.S. Department of Health and Human Services FaDA et al., 2023). An Investigational New Drug (IND) application must be submitted before initiating human trials, containing all preclinical data, manufacturing information, and clinical protocol (Holbein, 2009).

### Product-specific regulatory considerations

For LNP-mRNA products, the regulatory pathway benefits from precedents established by COVID-19 vaccines (S et al., 2022). Key CMC (Chemistry, Manufacturing, and Controls) requirements include: characterization of lipid components (ionizable lipids, phospholipids, cholesterol, PEG-lipids), quality control for mRNA purity and integrity, assessment of lipid impurity profiles, and demonstration of batch-to-batch consistency. Nonclinical safety assessments must address LNP biodistribution (with particular attention to gonadal accumulation), immunogenicity (including cytokine profiling and assessment of anti-PEG antibodies), and reproductive toxicity (U.S. Department of Health and Human Services FaDA et al., 2023). Given the testicular route of administration, regulators will expect evidence that the LNP formulation does not cause chronic inflammation or disruption of the testicular immune-privileged environment.

For AAV-based gene editing products, regulatory requirements are more extensive. In addition to the CMC requirements for vector production (including characterization of the AAV serotype, genome integrity, and empty/full particle ratios), regulators require: comprehensive biodistribution studies demonstrating minimal or no germline exposure; assessment of pre-existing humoral immunity and strategies to overcome neutralization; evaluation of vector shedding; and long-term monitoring for insertional mutagenesis and oncogene activation (Modell et al., 2025). The FDA’s draft guidance on gene therapy for inherited disorders provides a framework, but the unique context of NOA, in which the treatment is intended to produce sperm for reproduction, necessitates even more stringent scrutiny of heritable risks. For HITI-based approaches, regulators will require extensive off-target analysis using orthogonal methods (GUIDE-seq, CIRCLE-seq, and targeted deep sequencing) to detect unintended genomic alterations (Tsai et al., 2015; Tsai et al., 2017).

For stem cell-based approaches (SSC transplantation, iPSC-derived gametes), the regulatory framework is even more complex, falling under the combined oversight of gene therapy and cell therapy regulations. Requirements include: characterization of cell identity, purity, potency, and sterility; assessment of tumorigenicity (particularly for iPSC-derived products); evaluation of epigenetic stability; and demonstration of functional germ cell competence. The potential for off-target effects from *ex vivo* gene editing must be assessed using whole-genome sequencing of the modified cells prior to transplantation. In the European Union, the European Medicines Agency (EMA) regulates gene therapy products through the Committee for Advanced Therapies (CAT) (Agency and Agency, 2020), and the Priority Medicines Initiative (PRIME) scheme offers enhanced support for products addressing unmet medical needs (MEdicines, 2024; Alaburde et al., 2025).

### Patient selection and preclinical data requirements

To date, no clinical trials have been registered or initiated for gene therapy specifically targeting NOA. The design of first-in-human trials must carefully consider patient selection criteria, appropriate dosing regimens (including the potential for repeated administration), and robust safety monitoring endpoints. A comprehensive preclinical data package must encompass: pharmacology studies (target engagement, dose-response, duration); toxicology studies focusing on reproductive organs and germline cells (Moffit et al., 2022); biodistribution assessments quantifying vector distribution to off-target tissues (U.S. Department of Health and Human Services FaDA et al., 2023); genotoxicity evaluation for integrating vectors or off-target editing (Bastone et al., 2023); and reproductive toxicology studies on fertility, embryonic development, and offspring (U.S. Department of Health and Human Services FaDA et al., 2023). Given the absence of clinical data, a phased approach—starting with small, open-label safety studies in well-characterized patient populations with specific monogenic defects—would be a prudent first step.

### Regulatory oversight, informed consent, and public engagement

The regulatory landscape for gene therapy has evolved considerably with approvals for inherited disorders, cancer, and other conditions (Delshad et al., 2025; Ginn et al., 2024; Modell et al., 2025). For NOA gene therapy, alongside scientific considerations, important ethical, legal, and social implications must be addressed (Sikiru et al., 2025). Within this framework, informed consent for first-in-human trials warrants particular attention due to potential germline effects and the sensitive reproductive context (Mulder et al., 2016). Participants must be clearly informed that alternatives, such as donor sperm and adoption, remain available, though emerging technologies such as stem cell-derived gametes may 1 day provide additional avenues, raising further ethical questions (Mulder et al., 2016). These considerations become more complex when pediatric translation is contemplated for prepubertal boys with genetic NOA (Goossens et al., 2020), given the inability to provide informed consent, potential effects on pubertal development, and long latency between treatment and attempted reproduction (Goossens et al., 2020). Equitable access concerns arise given likely high costs; ensuring access regardless of socioeconomic status will be essential to avoid exacerbating existing disparities (Chambers et al., 2013). Regulatory oversight must balance innovation with protecting participants and future generations, and international harmonization of standards for germline effects assessment would promote consistent evaluation. Finally, public engagement is essential to ensure development reflects societal values and addresses concerns (Adashi et al., 2020).

### Ethical considerations and a path forward

Gene editing in the testis raises profound ethical questions, particularly when germ cells are targeted. The heritable nature of introduced genetic changes distinguishes testicular from somatic gene therapy and has generated substantial debate (Cwik, 2020; Almeida and Ranisch, 2022). International consensus statements have generally urged caution, recommending that germline editing for reproductive purposes should not proceed until safety and efficacy concerns are adequately addressed (National Academies of Sciences E, 2017). However, these statements have primarily pertained to embryo or gamete editing aimed at establishing pregnancies. The editing of SSCs for NOA therapy—intended to produce sperm for the individual’s own reproduction—presents a more ethically ambiguous situation (Sikiru et al., 2025; Mulder et al., 2016). Key considerations include informed consent, given that patients must understand unknown long-term consequences and theoretical risks to future generations; safety and efficacy, requiring an exceptionally high threshold for clinical translation due to heritable effects; equity and access, as these technologies may exacerbate existing disparities in reproductive care; and robust regulatory oversight to ensure responsible development (National Academies of Sciences E, 2017; Charo, 2019; Baylis, 2019). Importantly, somatic cell editing directed at Sertoli cells can rescue NOA without detectable germline transmission, circumventing many of these ethical concerns (Zhang et al., 2025). By targeting the testicular niche rather than germ cells, this approach achieves therapeutic benefit while minimizing heritable modification risks.

## Safety considerations for NOA gene therapy

### Preclinical safety evaluation: from mRNA to CRISPR

Preclinical safety evaluation of mRNA-based NOA therapeutics must contend with unique challenges specific to testicular delivery and the risk of germline involvement. These encompass four key areas, such as biodistribution, immunogenicity, reproductive toxicity, and genomic integrity. With respect to biodistribution, the tissue distribution of LNP-formulated mRNA and lipid components should be systematically evaluated, with special attention to gonadal accumulation and germline transmission risk (Hou et al., 2021). Regarding immunogenicity, TLR-mediated recognition can trigger inflammation that impairs spermatogenesis (Chen N. et al., 2017); the Pdha2 study addressed this by using m1Ψ-modified mRNA, which histologically showed no inflammatory infiltration or tissue damage (Mashiko et al., 2025). In terms of reproductive toxicity, studies must assess effects on fertility, embryonic and postnatal development (U.S. Department of Health and Human Services FaDA et al., 2023); the demonstrated fertility of treated mouse offspring provides important preliminary evidence against heritable effects, though larger animal models are still needed (Maynard et al., 2021). Lastly, genomic integrity was evaluated via whole-genome sequencing of offspring, which detected no large-scale abnormalities (>1 Mb) (Mashiko et al., 2025), offering reassurance for transient mRNA expression, though smaller mutations or epigenetic changes cannot be excluded.

### Vector-specific safety considerations

Each delivery platform presents unique safety challenges. For AAV vectors, concerns include pre-existing humoral immunity in the human population, which can neutralize the vector and reduce efficacy (Egorova et al., 2025), as well as the potential for vector integration into the host genome, which, although rare, carries a risk of insertional mutagenesis and oncogene activation (Sabatino et al., 2022). For LNP-mRNA formulations, the lipid components themselves can be immunogenic, and repeated administration may trigger an adaptive immune response that reduces therapeutic efficacy or causes local inflammation, potentially disrupting the testicular microenvironment (Chen K. et al., 2025; Jiang et al., 2025). For lentiviral vectors, the risk of insertional mutagenesis is more significant due to their propensity for integration into actively transcribed genes, raising serious safety concerns, particularly for germline applications (Kaiser et al., 2026; Schambach et al., 2013). A comprehensive preclinical safety package must therefore address these vector-specific risks using orthogonal assays.

For CRISPR-based approaches, off-target editing, defined as the introduction of unintended mutations at genomic sites with sequence similarity to the intended target, represents a major safety concern, as such effects in testicular gene editing could introduce germline mutations transmissible to offspring with unpredictable consequences (Fu et al., 2013; Pattanayak et al., 2013). Comprehensive off-target analysis is therefore essential, encompassing computational prediction followed by targeted deep sequencing alongside unbiased methods such as GUIDE-seq or CIRCLE-seq to identify off-target sites without *a priori* prediction (Tsai et al., 2015; Tsai et al., 2017). Beyond off-target editing, persistent Cas9 expression can induce p53 pathway activation and DNA damage accumulation even in the absence of sgRNA, potentially selecting for p53-inactivating mutations that compromise genomic integrity (Kosicki et al., 2018; Haapaniemi et al., 2018). Strategies to minimize these risks include transient delivery of Cas9 as mRNA or protein rather than persistent viral vector expression, alongside optimized sgRNA design to maximize on-target specificity (Doench et al., 2016).

Given that the ultimate goal of NOA therapy is to produce functional sperm for conception, reproductive toxicology studies must extend beyond the treated animal to include comprehensive assessment of the resulting offspring. This includes multi-generational monitoring to detect any potential heritable genetic or epigenetic effects, as well as assessment of developmental, behavioral, and reproductive outcomes in the F1 and F2 generations. Such studies are essential to establish that the transient presence of mRNA or the persistent effects of gene editing do not inadvertently compromise the health of future generations.

### Cost-effectiveness and equitable access

The development of gene therapy for NOA, like many advanced therapies, is likely to be costly, potentially limiting access to patients in high-income settings or those with comprehensive insurance coverage. The costs associated with vector production, quality control, preclinical testing, and regulatory compliance are substantial. As these therapies progress toward the clinic, it will be essential to consider strategies to reduce manufacturing costs and to develop reimbursement models that ensure equitable access, regardless of socioeconomic status. Failure to address these issues could exacerbate existing disparities in reproductive healthcare.

## Future directions

Current gene therapies for NOA have focused on a limited number of well-characterized targets. Expanding this repertoire will require continued gene discovery and functional validation of candidate variants. Large-scale sequencing initiatives hold promise for systematically identifying rare NOA-associated variants, while CRISPR-based functional studies in cell lines and animal models will be essential for establishing pathogenicity and elucidating disease mechanisms. For each newly implicated gene, the optimal strategy—whether mRNA replacement, gene editing, or alternative approaches—must be determined based on expression pattern, developmental timing, and mechanistic context.

However, several critical knowledge gaps must be addressed before clinical translation can proceed. Whether the inherently transient expression of mRNA therapy can be effectively synchronized with the protracted human spermatogenic cycle—or whether alternative dosing regimens or self-amplifying RNA platforms will be required—remains a critical unanswered question, and the immunogenic and safety implications of repeated intratesticular injections must be carefully evaluated.

Delivery efficiency represents another major hurdle: how can the current 55% tubular coverage achieved in mice be improved to a clinically meaningful level in the larger human testis, and can non-invasive systemic delivery be achieved, or will localized injection remain the only viable route? Long-term safety considerations are equally critical, as the consequences of even transient inflammation in the testicular niche on the epigenetic landscape of resulting spermatozoa remain unknown, and the risk of off-target mutations accumulating over time with gene editing approaches requires effective monitoring strategies. The role of testicular immune privilege in modulating immune responses to viral vectors or LNPs must be clarified, specifically whether this unique environment can be exploited to reduce immunogenicity or whether it will be overwhelmed by repeated dosing. Finally, beyond genetic diagnosis, the identification of biomarkers or testicular histology features that can predict patient responsiveness to specific gene therapy strategies will be essential for patient stratification.

Additionally, a structured assessment of the technology readiness level (TRL) for each approach would be valuable. Currently, mRNA replacement and somatic gene editing in Sertoli cells are at TRL 4-5 (validated in relevant animal models), while SSC transplantation and artificial gametes are at TRL 3-4 (proof-of-concept in animal models). In contrast, human clinical trials for NOA gene therapy remain at TRL 1-2 (basic research). This assessment highlights that, despite the promise, most strategies are still in early stages of development.

Addressing these questions will require a coordinated effort involving basic scientists, clinician-scientists, regulatory bodies, and patient advocacy groups. The coming decade will be pivotal in determining whether the promise of gene therapy for NOA can be realized in the clinic.

## Conclusion

Preclinical studies using mRNA replacement and Sertoli cell gene editing have restored spermatogenesis in genetic NOA mouse models, establishing compelling proof-of-concept for gene therapy in genetically defined NOA. However, translating these findings into clinical practice is contingent upon overcoming several substantial hurdles, including expanded gene targets, optimized testicular delivery, robust preclinical safety data (particularly germline effects), long-term safety validation, regulatory frameworks for first-in-human trials, and attention to ethical, legal, and social implications. With targeted therapies poised to restore endogenous spermatogenesis in genetically defined NOA patients, the coming decade may turn this vision of biological parenthood into reality, offering renewed hope to millions once devoid of therapeutic options.

## Glossary

AAV: Adeno-Associated Virus
AR: Androgen Receptor
ARCA: Anti-reverse Cap Analog
ARRIVE: Animal Research Reporting of *In Vivo* Experiments
ART: Assisted Reproductive Technologies
BTB: Blood-testis Barrier
CAR: Coxsackievirus and Adenovirus Receptor
CAT: Committee for Advanced Therapies
CBER: Center for Biologics Evaluation and Research
CLDN11: Claudin-11
CMC: Chemistry, Manufacturing, and Controls
DSBs: Double-strand Breaks
EMA: European Medicines Agency
ESCs: Embryonic Stem Cells
FDA: Food and Drug Administration
FSHR: FSH Receptor
GMP: Good Manufacturing Practice
HDR: Homology-directed Repair
HITI: Homology-independent Targeted Integration
ICSI: Intracytoplasmic Sperm Injection
IND: Investigational New Drug
iPSCs: Pluripotent Stem Cells
IVS: *In vitro* Synthesis
kb: kilobases.
KO: Knockout
LNP: Lipid Nanoparticle
micro-TESE: Microdissection Testicular Sperm Extraction
NGS: Next-generation Sequencing
NHEJ: Non-homologous End Joining
NHPs: Non-human Primates
NOA: Non-obstructive Azoospermia
OA: Obstructive Azoospermia
OMIM: Online Mendelian Inheritance in Man
*PDHA*: Pyruvate Dehydrogenase E1 Alpha
PGCLCs: Primordial Germ Cell-like Cells
PRIME: Priority Medicines Initiative
*RIG-I*: Retinoic Acid-inducible Gene I
SC: Synaptonemal Complex
SRRs: Sperm Retrieval Rates
SSCs: Spermatogonial Stem Cells
*TEX11*: Testis-expressed 11
TLRs: Toll-like Receptors
TRL: Technology Readiness Level
UTRs: Untranslated Regions
